# Web based application for Casemix Grouper (Online Casemix Grouper)

**DOI:** 10.1186/1471-2458-14-S1-P15

**Published:** 2014-01-29

**Authors:** Hasrul Reeza Mustaffa, Syed Mohamed Aljunid, Zafar Ahmed, Syed Mohamad Hamzah Al-Junid

**Affiliations:** 1 United Nations University-International Institute for Global Health, UKMMC Complex, Jalan Yaacob Latiff, Bandar Tun Razak, 56000 Cheras, Kuala Lumpur, Malaysia; 2 Jabatan Kesihatan Masyarakat, Pusat Perubatan Universiti Kebangsaan Malaysia, Jalan Yaacob Latif, Bandar Tun Razak, 56000 Cheras, Kuala Lumpur, Malaysia

## Background

Casemix Grouper software is a tool to improve efficiency and quality of care in hospitals and medical centres. The utilisation of Casemix Grouper among hospitals is very limited and depends on their existing local machine and hardware. While Casemix Grouper provides a crucial output in the form of Casemix groups, the hardware machine should not be a limiting factor to hospital. Thus, the objective of this development was to provide the utility for end user to use the Casemix Grouper on web environment.

## Materials and methods

The conventional way of providing Casemix Grouper service is by deploying standalone software at user’s local machine. This will create problem when user’s machine are diverse and does not meet the requirement for running the Casemix Grouper. The online Casemix Grouper will have 2 modules, which are for user and for the admin. The user module includes the authentication login and then uploading the input file. Developing a web based using Java Servlet Page (JSP) will provide access to all the available browsers on any operating system, so that end user can upload the Casemix input file. After the input file has been uploaded, online grouper will process the input file and generate an output file. The user will receive the output file via user-registered email. The admin module will also have authentication login and will be able to monitor the whole process for each users.

## Conclusions

With the implementation of Casemix Grouper as a web-based application, user can now avoid the stringent hardware requirement on their local computers for standalone Casemix Grouper. The end users can now focus only on generating the input file and will receive output file without extra effort. This usage of web application of Casemix Grouper software will facilitate the provision of efficient and quality care in hospitals and medical centres.

